# The effects of aluminium on plant growth in a temperate and deciduous aluminium accumulating species

**DOI:** 10.1093/aobpla/plw065

**Published:** 2016-10-26

**Authors:** Marco Schmitt, Toshihiro Watanabe, Steven Jansen

**Affiliations:** 1 Institute of Systematic Botany and Ecology, Ulm University, Albert-Einstein-Allee 11, 89081 Ulm, Germany; 2 Research Faculty of Agriculture, Hokkaido University, Kita-9, Nishi-9, Kitaku, 060-8589 Sapporo, Japan

**Keywords:** Accumulation, aluminium, aluminon, hydroponics, pyrocatechol-violet, Symplocos paniculata, Symplocaceae

## Abstract

Aluminium (Al) is a phytotoxic element affecting the growth and yield of many crop plants, especially in the tropics. Yet, some plants are able to accumulate high levels of Al. The monogeneric family Symplocaceae represents an Al accumulating family including many tropical and evergreen species with high Al levels in their above ground plant tissues. It is unclear, however, whether Al accumulation also characterises temperate species of *Symplocos*, and whether or not the uptake has a beneficial growth effect. Here, we investigate if the temperate, deciduous species *Symplocos paniculata* is able to accumulate Al by growing seedlings and saplings in a hydroponic setup at pH 4 with and without Al. Pyrocatechol-violet (PCV) and aluminon staining was performed to visualize Al accumulation in various plant tissues. Both seedlings and saplings accumulate Al in their tissues if available. Mean Al levels in leaves were 4107 (±1474 mg kg^−1^) and 4290 (±4025 mg kg^−1^) for the seedlings and saplings, respectively. The saplings treated without Al showed a high mortality rate unlike the Al accumulating ones. The seedlings, however, showed no difference in growth and vitality between the two treatments. The saplings treated with Al showed new twig, leaf and root development, resulting in a considerable biomass increase. PCV and aluminon staining indicated the presence of Al in leaf, wood and bark tissue of the plants. *S. paniculata* shares the capacity to accumulate Al with its tropical sister species and is suggested to be a facultative accumulator. Whether or not Al has a beneficial effect remains unclear, due to developmental differences between seedlings and saplings. Al is suggested to be transported via the xylem transport system into the leaves, which show the highest Al levels. Radial transport via ray parenchyma to bark tissue is also likely given the high Al concentrations in the bark tissue.

## Abstract (precis)

Although aluminium (Al) is toxic for the vast majority of angiosperm plants, high concentrations of Al (i.e., < 1,000 mg·kg-1 dry mass) are found in some plants. Here, we investigate the Al accumulation behaviour in the temperate, deciduous species Symplocos paniculata, which belongs to a mainly tropical genus known to accumulate high levels of Al in its aboveground tissues. Based on a growing experiment in hydroponics with and without Al, we show that S. paniculata has the capacity to accumulate Al and that the absence of Al in the nutrient solution has a negative impact on the performance of saplings.

## Introduction

Although abundantly present in all terrestrial biomes, aluminium (Al) is typically absent as nutrient and as trace element within biochemical pathways of the living biosphere (Pogue and Lukiw, 2014). It is considered to be phytotoxic to the majority of plants if the soil pH decreases below 5.5 (Delhaize and Ryan, 1995; von Uexküll and Mutert, 1995), which causes Al to become soluble while changing its hydroxide form Al(OH)_3_ to toxic forms such as Al(OH)_2_^+^, Al(OH)^2+ ^and Al^3+ ^(Kinraide, 1991, 1997). Immediate responses of plants sensitive to Al exposure include cease of root growth, lesions of the root tissue and subsequent nutrient deficiencies due to impaired uptake (Ryan *et al.*, 1994; Kochian *et al.*, 2004; Yang *et al.*, 2011; Kopittke *et al.*, 2015). The majority of plants growing in acidic soils have developed various strategies to avoid the uptake of Al into their plant body. Known strategies are the increase of the soil pH to prevent the solubility of toxic Al^3+ ^(Taylor, 1991), or the exudation and complexation of Al through various organic compounds (e.g. oxalate, malate, catechins or phenolics) at the root level (Taylor, 1991; Barceló and Poschenrieder, 2002; Watanabe *et al.*, 2008). Some plants are known to accumulate more than 1000 mg kg^−^^1^ drymass of Al in their stems and leaves and are often referred to as Al-accumulators (Jansen *et al.*, 2002; Haridasan, 2008). Al accumulating species are mainly found in tropical regions and are, with few exceptions, woody plants most of which also possess blue flowers and/or fruits (Hutchinson and Wollack, 1943; Chenery, 1948a, b , 1949). Physiological mechanisms underlying the uptake of Al into aboveground plant tissues have been investigated in *Fagopyrum esculentum* (Buckwheat), *Melastoma malabathricum, Hydrangea macrophylla* and *Camellia sinensis* (tea), but remain unclear in many plant groups (Ma *et al.*, 1997a, b; Shen *et al.* 2002; Watanabe *et al.*, 2005b; Fung *et al.*, 2008; Hajiboland *et al.*, 2013; Wang *et al.*, 2015).

The uptake of Al from the soil into the plant body does not lead to an equal distribution within the above ground plant organs. Al is suggested to follow the transpiration stream through the xylem into the leaf mesophyll of the plant, where the highest concentrations can be found (Haridasan *et al.*, 1986; Tolrà *et al.*, 2011; Maejima *et al.*, 2014; Schmitt *et al.*, 2016). High concentrations of Al in the bark also indicate a potential transport pathway via the phloem tissue (Zeng *et al.*, 2013; Schmitt *et al.*, 2016). However, the mechanisms underlying the chemical detoxification within Al accumulating species remain poorly understood. Possible mechanisms observed in *Fagopyrum* and *Hydrangea* include secretion and chelation of Al through organic acids such as oxalic acid or citrate (Ma *et al.*, 1997a, b; Nguyen *et al.*, 2003). While Al accumulating angiosperms are rather rare, representing about 5 % of all angiosperm species, this feature is characteristic of various monophyletic plant groups (Jansen *et al.*, 2002).

In some species such as *Camellia sinensis* and *Melastoma malabathricum*, the availability of Al has a beneficial effect on plant growth by improving the growth of roots and the nutrient and water uptake capacity (Watanabe *et al.*, 2005a; Hajiboland *et al.*, 2013). Generally, two different types of accumulators can be defined, namely, obligate and facultative. Obligate accumulators can only grow on metalliferous soils and are unable to survive if a particular element is unavailable. Facultative accumulators, however, are growing well regardless of whether the soil contains a given metal or not (Pollard *et al.*, 2014). Applying these two terms to Al accumulators, however, is not straightforward because growth experiments with Al have been conducted for a few species only. No difference in plant growth was observed for *Fagopyrum*, *Camellia* and *Hydrangea*, indicating that these species might be facultative accumulators (Ma *et al.*, 1997a; Ma and Hiradate, 2000; Tolrà *et al.*, 2011). Obligate Al accumulation was reported for *Miconia albicans*: seedlings of this Melastomataceae species growing in the Brazilian cerrado show a pronounced chlorosis and die-back when growing in a calcareous soil with alkaline pH and no available Al (Haridasan, 1988).

*Symplocos* includes ca. 300 species of woody trees and shrubs (Fritsch *et al.*, 2008). The monogeneric family Symplocaceae is suggested to originate in Eurasia and has from there dispersed to the American continent (Fritsch *et al.*, 2015). *Symplocos* is most commonly found in montane tropical and subtropical rainforests, but also present in low altitude, temperate regions in Eurasia and the Americas, where is it also cultivated as ornamental shrub with dark blue berries (Doney, 1945; Nooteboom, 1977; Wang *et al.*, 2004; Fritsch *et al.*, 2008). The natural distribution of *Symplocos paniculata* is on slopes in mixed forests above 800 m across Taiwan, China, Japan, Korea and India (Rong-fen and Nooteboom, 1996). Within the genus, *S. paniculata*, *S. chinensis* and *S. tinctoria* are the only *Symplocos* species with deciduous leaves (Nooteboom, 1977; Rong-fen and Nooteboom, 1996; Fritsch *et al.*, 2008). Furthermore, according to recent phylogenetic analyses, two of these species (*S. paniculata* and *S. chinensis*) represent a sister clade to all other *Symplocos* species (Wang *et al.*, 2004; Fritsch *et al.*, 2008).

*Symplocos* is known to include many strong Al accumulators, as reported for three *Symplocos* species (*S. ophirensis*, *S. odoratissima*, *S. ambangensis*) from Sulawesi, with an average Al concentration of 24180 ± 7236 mg kg^−^^1^ in old leaves (Schmitt *et al.*, 2016). Maejima *et al.* (2014) reported Al accumulation in *S. chinensis*, growing in an Al containing hydroponic setup (8309 ± 7236 mg kg^−^^1^). The highest leaf level of Al in *Symplocos* species was recorded in leaves of *S. spicata* with 72300 mg kg^−^^1^ (von Faber, 1925). Some *Symplocos* species are of economic interest to traditional weaving communities across the Indonesian archipelago, where Al containing leaves are used as a mordant in the natural dying process (Jansen *et al.*, 2002; Cunningham *et al.*, 2011; Schmitt *et al.*, 2016).

In this study, we investigated the Al uptake in the temperate, deciduous species *Symplocos paniculata* (Symplocaceae) using a hydroponic setup. We hypothesize that *S. paniculata* has a similar Al accumulation capacity as the evergreen, (sub-)tropical species. Earlier, semi-quantitative tests performed on *Symplocos*, show that 141 specimens out of 142 tested ones were Al accumulating as summarized in Jansen *et al.* (2002). Alternatively, it is possible that Al accumulation has been lost or remains poorly developed in *S. paniculata* because this species takes an isolated phylogenetic position within the genus, together with its temperate and deciduous habitus. Furthermore, we investigated whether this species shows similar distribution patterns of Al in its plant organs as values recently reported in tropical evergreen species (Schmitt *et al.*, 2016). If this would be correct, the highest Al levels would occur in its leaves, followed by bark tissue and wood. Staining of anatomical sections was applied to visualize the distribution of Al in aboveground organs and tissue (Chenery, 1948b; Watanabe *et al.*, 1998; González-Santana *et al.*, 2012). By performing a growth experiment with and without Al we also tested if Al has a beneficial effect on its growth (Hajiboland *et al.*, 2013), which can be expected based on the close phylogenetic relationship between Symplocaceae and Theaceae.

## Methods

### Plant material

We obtained 16 potted plants of *Symplocos paniculata* from a nursery (Rein&Mark Bulk, Boskoop, The Netherlands), which will be referred to hereafter as ‘saplings’. The nursery grew the plants from seeds obtained from the UK. The saplings were ca. 1–3 years old and 15–50 cm tall. The pH of the soil was ca. 5.5.

In addition, 20 seedlings of *S. paniculata* were grown from seeds obtained from the Charles Keith Arboretum in North Carolina, USA in autumn 2014. The pulp of the fruit was removed and seeds were sown in sand in September 2014. The seeds were stored at 4 °C for 3 months and then placed outside at ambient temperature in January 2015. When the first leaves were visible in May 2015, seedlings were put into soil (bog soil with pumice, pH ca. 5.5) prior to a hydroponic experiment. A total of 20 seedlings (5–10 cm tall) were grown outside at the Botanical Garden of Ulm University in half-shaded conditions until the hydroponic experiment started in August 2015. These plants are hereafter referred to as ‘seedlings’.

### Hydroponic experiments

The saplings were divided into two groups (i.e. *n*  = 8 individuals per group), each group having the same number of specimens with a similar height and age. First, the soil was carefully removed from the plants with tap water. After removal of the substrate the roots were thoroughly rinsed with demineralized water. Plants were weighted and transferred into 35 l vessels, with four plants per vessel, respectively. The vessels contained a standard nutrient solution following Watanabe and Osaki (2001) and were constantly aerated with an aquarium pump (EHEIM air pump 200, EHEIM GmbH & Co KG, Deizisau, Germany). The solution consisted of 2.14 mM N (NH_4_NO_3_), 32 μM P (NaH_2_PO_4_·2H_2_O), 0.77 mM K (K_2_SO_4_: KCl = 1:1), 1.25 mM Ca (CaCl_2_·2H_2_O), 0.82 mM Mg (MgSO_4_·7H_2_O), 35.8 μM Fe (FeSO_4_·7H_2_O), 9.1 μM Mn (MnSO_4_·4H_2_O), 46.3 μM B (H_3_BO_3_), 3.1 μM Zn (ZnSO_4_·7H_2_O), 0.16 μM Cu (CuSO_4_·5H_2_O) and 0.05 μM Mo ((NH_4_)_6_Mo_7_O_24_ ·4H_2_O). The pH was adjusted to pH 4 with HCl and NaOH using a pH test stick (pH PAL Plus, ETI Ltd, UK). The nutrient solutions were changed on a weekly basis. The plants were kept in this reference solution for 2 weeks to adapt to the hydroponic environment. Then, two vessels including a total of eight plants were treated with the standard nutrient solution with added 1 mM L^−^^1^ AlCl_3_ until the end of the experiment (hereafter named +Al), whereas the other two vessels were not given any Al (−Al) **[see Supporting Information]**. Furthermore, the plants were sprayed with demineralized water on a daily basis to avoid dehydration and to reduce the heat stress by sun exposure at midday. During the third week all vessels were covered with a grey lid to prevent the growth of algae in the nutrient solution **[see Supporting Information]**. At the end of the experiment, all plants (including the fallen leaves) were removed from the vessels, weighed again, rinsed with demineralized water, put into plastic bags and kept in the freezer at −25 °C for further analysis.

The experimental conditions of the seedling experiment in 2015 were analogous to the hydroponic setup with saplings in 2014 except for minor differences. Due to the smaller plant size of the seedlings, we used a vessel size of 4.5 l. Each vessel contained five plants, with four vessels in total (*n*_plants_  = 20 individuals). Two vessels were treated with the standard nutrient solution with 1 mM AlCl_3_ after 2 weeks of acclimatisation in the standard nutrient solution. At the end of the experimental phase, both the roots and leaves of the 20 saplings were scanned (EPSON Expression 10 000 XL, SEIKO EPSON Corporation, Nagano, Japan). Root morphological parameters were analysed with WinRhizo 2012 and leaf area was measured using ImageJ (Version 1.49, U. S. National Institutes of Health, Bethesda, Maryland, USA, http://imagej.nih.gov/ij/ (26 September 2016), 1997–2015). Both experiments were conducted inside the greenhouses of the Botanical Garden Ulm. Climate conditions were controlled to avoid considerable variation. Temperatures were kept above 12 °C at night and below 33 °C during the day, with a relative humidity between 33 % and 94 %. The plants were never fully sun exposed due to a shading system on the roof of the greenhouse.

### Elemental analysis

**Figure 1:**
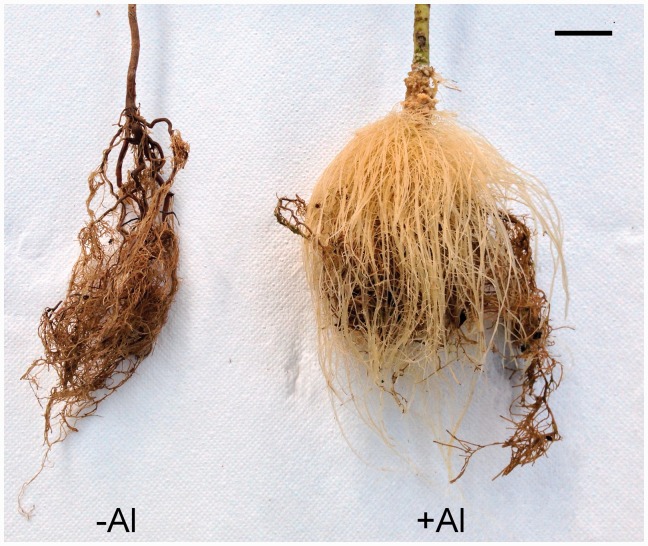
The roots of *Symplocos paniculata* saplings after growing for 17 weeks in hydroponics. The +Al plants grew new white roots, while these were clearly absent in –Al plants. In the +Al treatment, 1 mM AlCl_3_ was added to the standard nutrient solution (pH 4), but no Al was available to the –Al plants. Scale bar = 2 cm.

Each specimen was separated into leaves, bark, wood and roots, which were analysed separately. Root material was defined as the sum of tissue that was immersed in the nutrient solution. Although no differentiation between bark and wood tissue of the roots was made, the new roots of the +Al treatments were separated from the old roots and could easily be distinguished by their white colour (Fig. 1). The tissues were put into paper bags and oven dried at 75 °C for 24 h. Samples were then ground with a centrifuge mill (ZM1, Retsch, Germany); 50 mg of the sample was put into 10 ml PTFE vessels, and digested with HNO_3_ in a microwave oven (Mars 5 plus, CEM, Germany) at 200 °C for 30 min. Digestions were diluted to 25 ml with a 0.25 % (w/v) CsCl-solution as ionic buffer. The concentrations of Al, K, Ca, Mg and Fe were measured based on microwave-plasm atomic emission spectrometry (MP-AES, Agilent 4100, Agilent Technologies, Australia). Before starting the hydroponic experiment, Al concentrations in leaves of the saplings were measured to determine the pre-treatment concentration of Al in the plants, which was 390 mg kg^−^^1^ in the leaf tissue. Furthermore, the Al concentration of 25 seeds and their fleshy pericarp was 3450 mg kg^−^^1^ and 6150 mg kg^−^^1^, respectively.

In the sapling experiment (*n* = 16 specimens), 12 wood samples, 16 leaf samples, 9 bark samples, 16 old root samples and 6 new root samples were analysed. The seedling experiment (*n* = 20 specimens) included a total of 18 bark, 20 leaf, 20 root and 18 wood samples. Hence, all plants, including those that died, were used for our chemical analyses. We were unable to separate the bark and wood tissue for four sapling specimens and two seedling specimens, which were not included in our analyses.

### Biomass and root–shoot ratio

The plants were weighed before they were put into the vessels at the beginning of the experiment. At the end of the experiment, the total weight of each plant was measured again. All leaves that were shed during the experimental phase were collected and included in the biomass calculation. As a relative measure, gain or loss of fresh biomass (Δm) was defined as:

(1) $$△m\;={\;m}_{\text{after}}–{\;m}_{\text{before}}$$

with *m*_after_ representing the plant weight in g of the whole plant at the end of the experiment, and *m*_before_ the plant mass before the experiment. The root–shoot ratio was calculated by dividing the dry mass of root tissue by the sum of dried above-ground tissues (i.e. leaves, bark and wood) for each plant. Both root–shoot ratio and Δ*m* were calculated for all specimens in both experiments (saplings: *n* = 16; seedlings: *n* = 20).

### Visualisation of Al accumulating tissue

For the visualisation of Al in plant tissue, a total of eight saplings, four from each treatment, were sampled. Wood sections were cut with a sliding microtome. Leaf material was cut into 0.5 × 0.5 cm sections and fixed in FPA (10 % formalin [37 %], 5 % propionic acid, 50 % ethanol [95 %] and 35 % distilled water) over night. Samples were then dehydrated using a graded series of ethanol (30, 50, 70 and 96 %) and *tert*-butanol, and embedded in paraffin. The embedded leaf sections were cut with a rotary microtome, transferred on adhesive-coated objective slides (Menzel-Gläser, Thermo Scientific, Gerhard Menzel GmbH, Braunschweig, Germany) and oven-dried at 30 °C overnight. The paraffin was then washed out using Neo-Clear (Merck KGaA, Darmstadt, Germany) and a graded ethanol series (95, 70, 50 and 30 %). Two staining methods were applied to locate high levels of Al in the tissue sections. Staining was conducted using a 0.052 % (w/v) pyrocatechol-violet (PCV) solution in 2.5 % hexamine–NH_4_OH buffer (pH 6.2), which was applied for 15 min and then washed with the buffer solution prior to mounting (Watanabe *et al.*, 1998). The second staining included ammonium aurin tricarboxylate (aluminon) (Chenery, 1948b; Clark and Krueger, 1985). After staining, the sections were washed in an additional ethanol series and mounted in Neo-Mount (Merck KGaA, Darmstadt, Germany).

### Statistical analysis

A Shapiro–Wilk test was performed to check for normal distribution and to determine the statistical tests to be applied. Differences between group means were analysed using the non-parametric Wilcoxon rank-sum test (Δbiomass, [Al], [Ca], [Fe]_roots_) or Welch’s *t*-test for parametric data (root–shoot ratio). Correlations between Al and calcium (Ca) were computed using the *psych* package of R with Spearman’s rho for non-parametric data. All statistical analyses were carried out with the freeware ‘R’ (RStudio, Version 0.99.902, RStudio Inc., Boston, USA).

## Results

### Hydroponic experiments on saplings and seedlings

After putting the saplings in the hydroponic solution for two weeks during the pre-treatment period, one sapling in the –Al treatment was considered dead according to visual properties (i.e. leaf desiccation and strong chlorosis) and showed no signs of recovery afterwards. A similar sapling was found in the +Al treatment, but was found to recover two weeks later. Regardless of the size of the plant and the +Al or –Al treatment, some leaves showed yellowish-brown leaf margins after 3 weeks (i.e. 2 weeks of pre-treatment and 1 week of +Al or –Al treatment). In the –Al treatment, basal leaves started to dry out without changing their colour. Two specimens from the +Al treatment showed a similar condition after the second week (i.e. before the +Al treatment started). In week 4, all saplings of the –Al treatment were seriously stressed and showed clear signs of desiccation. After week 5 (i.e. 3 weeks after starting with the Al treatment), new white roots were visible in the +Al saplings, which continued growing until the end of the experiment (Fig. 1). After 17 weeks of treatment, all plants in the –Al treatment were considered to be dead, with only the largest sapling remaining in an unhealthy, stressed condition. Except for two plants, all specimens in the +Al treatment developed new side branches and leaves and were healthy **[see Supporting Information]**.

**Figure 2:**
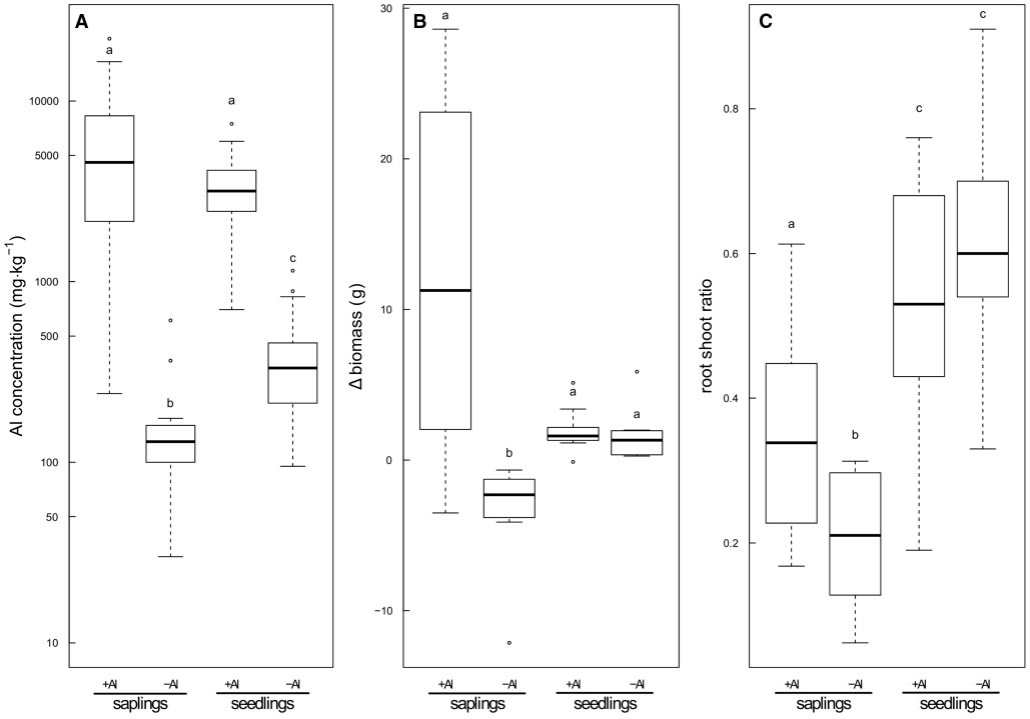
(A) Boxplot showing the aluminium concentration in saplings (*n* = 16) and seedlings (*n* = 20) of *Symplocos paniculata* at the entire plant level. All plants were grown in hydroponics for a minimum of 2 months. The +Al condition had a 1 mM AlCl_3_ solution added to the standard nutrient solution, while no Al was available to the –Al plants. (B) Relative change in biomass defined as the difference between the total biomass before and after the hydroponic experiment. (C) Root–shoot ratio of the two experiments. *n*_saplings_ = 8 specimens per treatment, *n*_seedlings_ = 10 specimens per treatment. Different letters indicate significant in a pairwise comparison of each group (*P* < 0.05; A, B = Wilcoxon rank-sum test; C = Welch’s *t*-test).

Changes in the fresh biomass during the hydroponic experiment showed a clear difference between the two treatments (Fig. 2). All –Al saplings had negative values with an average loss in fresh biomass of −3.45 g ± 3.46 g, whereas the +Al treatment showed a gain of 12.24 g ± 11.24 g. The highest loss was −12.14 g, and the largest increase was 28.62 g. A significant (Welch’s *t*-test, *t*  = 2.3231, df  = 11.718, *P*  = 0.03903) difference was found between the root–shoot ratio of the –Al treatment (0.20 ± 0.08) and the +Al treatment (0.35 ± 0.14, Fig. 2).

**Figure 3:**
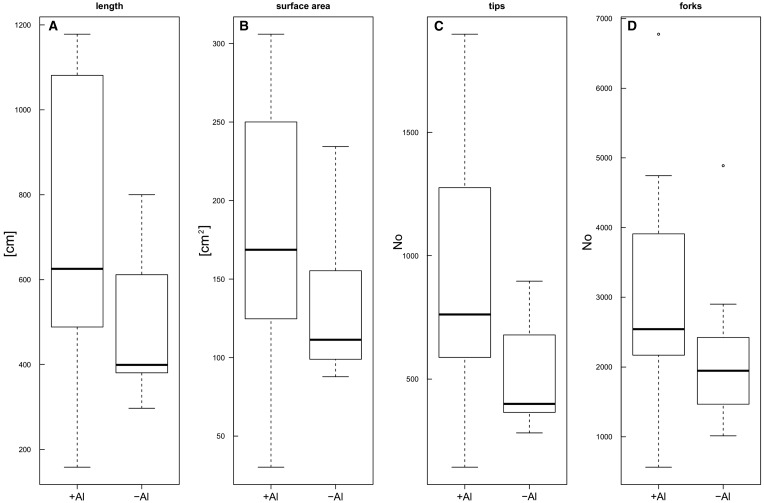
Root morphological features of the *Symplocos paniculata* seedlings, analysed with WinRhizo 2012. The +Al condition had a 1 mM AlCl_3_ solution added to the standard nutrient solution, which was adjusted to pH 4, but no Al was available to the –Al plants. Shown is the sum of the root length (A), the calculated surface area of the roots (B), the number of root tips (C) and the number of forks (i.e. when a root segment bifurcates, D). None of the features showed a significant difference between the two treatments (Wilkoxon rank-sum test), although +Al levels were typically higher and more variable.

In contrast to the saplings, the seedlings did not show visible differences in vitality (e.g. leaf chlorosis, desiccation) between the +Al and −Al conditions. No dead seedlings were observed in both conditions, although several plants dropped a few leaves. Furthermore, no difference in biomass (Wilcoxon rank-sum test, W  = 62, *P*  = 0.393) and root-shoot ratio (Welch’s *t*-test, *t*  = −0.8788, df  = 17.78, *P*  = 0.3912) was found between the +Al and –Al seedlings (Fig. 2). The root morphology between the two conditions did not differ significantly from each other (Wilcoxon rank-sum test, root length: W  = 76, *P*  = 0.05243; root surface area: W  = 75, *P*  = 0.06301; root tips: W  = 71, *P*  = 0.123; root forks: W  = 71, *P*  = 0.123; Fig. 3), although the mean values of the root traits measured were higher in the +Al treatment than in the –Al seedlings.

### Al accumulation in the plant tissues

There was a significant difference (Wilcoxon rank-sum test, W  = 845, *P* < 0.01, Fig. 2) in Al concentration between the +Al saplings (5728 mg kg^−^^1 ^±^ ^4792 mg kg^−^^1^) and –Al saplings (145 mg kg^−^^1 ^±^ ^117 mg kg^−^^1^) at the entire plant level, summarizing all above and belowground organs. The highest Al concentrations in the +Al treatment were found in the new roots (12936 mg kg^−^^1  ^±^ ^5294 mg kg^−^^1^, see **Supporting Information**), whereas the stem wood tissue had the lowest concentration (1618 mg kg^−^^1 ^ ±^ ^842 mg kg^−^^1^). Highest Al concentrations in the –Al treatment were found in the root tissue (196 mg kg^−^^1 ^±^ ^168 mg kg^−^^1^) and the lowest in the stem wood (46 mg kg^−^^1 ^±^ ^21 mg kg^−^^1^), respectively.

**Figure 4:**
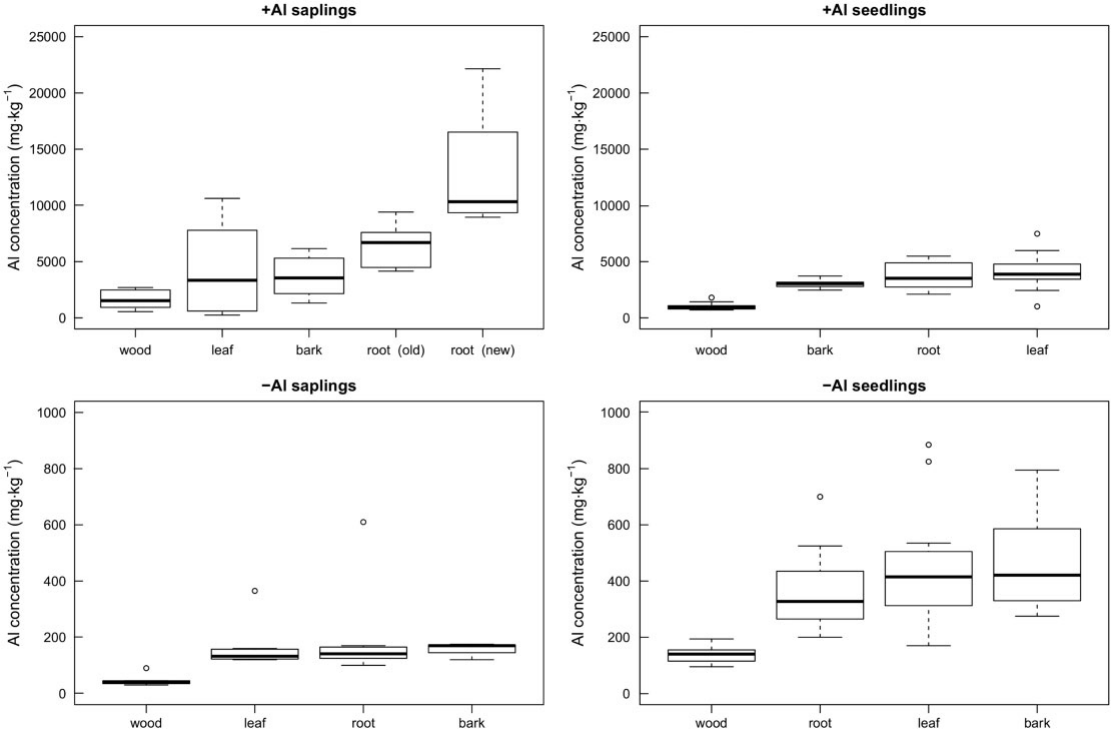
Comparison of Aluminium concentrations in various organs of *Symplocos paniculata* saplings and seedlings. All plants were grown in a hydroponic solution for a minimum of two months. The +Al condition had a 1 mM AlCl_3_ solution added to the standard nutrient solution with a pH adjusted to 4, and no Al was available to the –Al plants. New roots were only formed in the +Al sapling plants and not in the seedlings.

In the seedling experiment, the mean Al concentration of all tissues in the +Al treatment (3242 ± 1584 mg kg^−^^1^) was significantly higher (Wilcoxon rank-sum test, W  = 2235.5, *P* < 0.01) than the concentration in the –Al treatment (381 ± 226 mg kg^−^^1^, Fig. 2). In the +Al treatment, the highest Al levels were found in the leaves with a mean concentration of 4107 (± 1474) mg kg^−^^1^ dry mass (Fig. 4, see **Supporting Information**), followed by the root (3749 ± 1304 mg kg^−^^1^), bark (3054 ± 419 mg kg^−^^1^) and the wood tissue (1038 ± 357 mg kg^−^^1^), respectively. The Al values were overall lower in the –Al treatment and had the highest levels in the bark tissues (481 ± 197 mg kg^−^^1^). The leaf Al concentration in the –Al seedlings had a mean value of 456 (± 243) mg kg^−^^1^ and was slightly lower than the bark concentration. The lowest concentration was found in the wood tissue (136 ± 32 mg kg^−^^1^).

### Concentration of additional elements

The Ca concentration at the entire plant level in the saplings was found to be significantly lower (Wilcoxon rank-sum test, W  = 292.5, *P*  = 0.04288) in the +Al treatment (4205 ± 4007 mg kg^−^^1^) than in the –Al treatment (5069 ± 3623 mg kg^−^^1^) **[see Supporting Information]**. No significant correlation between Al and Ca was found (Spearman correlation, −Al: r_s_  = 0.23, *P*  = 0.27; +Al: r_s_  = −0.18, *P*  = 0.31). The K concentration in the sapling roots differed significantly (Wilcoxon rank-sum test, W  = 41, *P*  = 0.0293) between the two treatments when comparing the new roots of the +Al treatment with the roots of the –Al treatment. However, no significant difference in K was found when comparing the old roots of the +Al treatment with the roots of the –Al treatment (Wilcoxon rank-sum test, W  = 48, *P*  = 0.1049).

The concentrations of Ca and Mg were similar in both treatments for each plant organ of the seedlings. For both treatments, a significant, positive correlation was found between Al and Ca (Spearman correlation, −Al: *r*_s_  = 0.63, *P* < 0.01; +Al: *r*_s_  = 0.69, *P* < 0.01). The Fe concentration in the roots was significantly (Wilcoxon rank-sum test, W  = 10.5, *P* < 0.01) lower in the +Al treatment (2644 ± 1437 mg kg^−^^1^) than in the –Al condition (6668 ± 4242 mg kg^−^^1^, see **Supporting Information**).

### Al visualisation based on light microscopy

**Figure 5:**
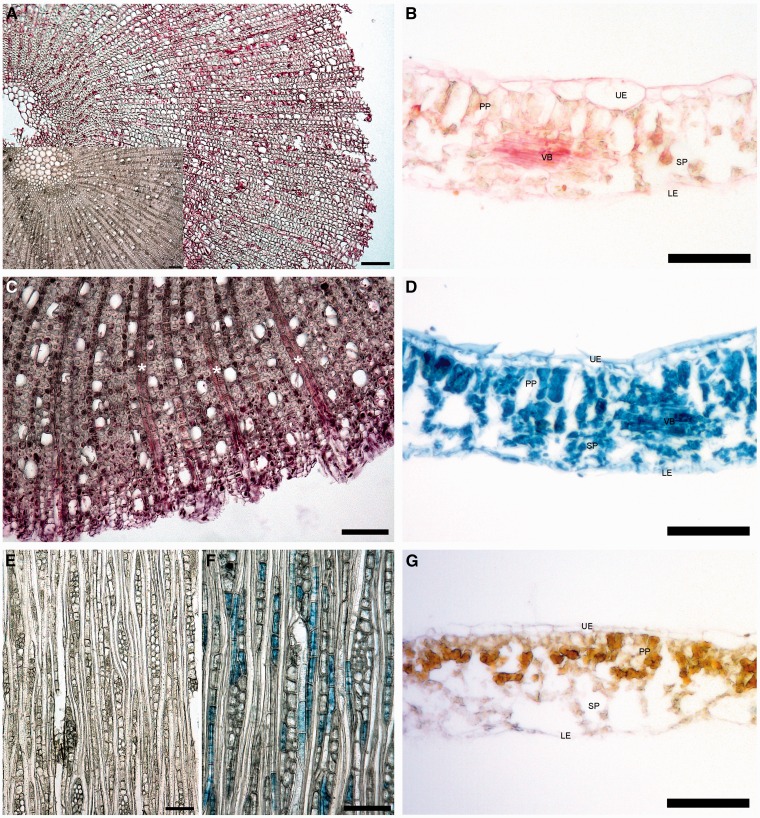
Leaf and wood cross sections of *Symplocos paniculata* saplings stained with aluminon or pyrocatechol-violet (PCV) after treatment with 1 mM AlCl_3_ (+Al treatment). (A) Stem cross section stained with aluminon. (B) Leaf cross section stained with aluminon. (C) Stem cross section stained with aluminon showing the presence of Al in the ray parenchyma (white asterisk). (D) Leaf cross section stained with PCV. (E) Tangential stem wood section of the –Al treatment stained with PCV. (F) Tangential stem wood section of the +Al treatment stained with PCV. (G) Leaf cross section of the –Al treatment after staining with aluminon. UE = upper epidermis, LE = lower epidermis, PP = palisade parenchyma, SP = spongy parenchyma, VB = vascular bundle. Scale bar = 100 µm.

Both staining solutions applied visualized Al in leaf tissues of the +Al plants, but not in the –Al plants (Fig. 5). PCV stained Al accumulating tissue in blue and was present in both the wood and the leaf tissue. In the wood tissue, tangential sections showed some blue stained ray parenchyma cells and axial parenchyma cells (Fig. 5). Cross-sections showed coloured cell walls in older growth rings and a weak blue colour in the pith parenchyma. In both tangential and cross sections, the bark tissue was blue. Colourless structures included most vessels, tracheids, and fibres in the younger wood. Leaf cross sections showed the presence of Al in the mesophyll, where the colour was stronger in the spongy parenchyma than in the palisade cells (Fig. 5). The vascular bundles of secondary and tertiary veins also contained Al, but no distinction between phloem and xylem could be made. The main vein, however, showed stained parenchyma cells in the xylem tissue and no colour in the vessels and tracheids within the vascular bundle **[see Supporting Information]**. Some cells within the phloem of the main vein were stained blue.

Similar results as for PCV were observed for aluminon, which in the presence of Al was creating a red crimson pigment. Red cells were found across the pith, wood and bark tissue (Fig. 5), but unstained tissue could not be clearly distinguished from coloured tissue. Tangential sections visualised Al in both radial and axial parenchyma cells, with a more pronounced signal than the PCV stained sections. Not all parenchyma cells in the tangential sections showed the same colour intensity. The leaf sections stained with Aluminon showed a pale red colour in the cell walls of the leaf epidermis, spongy parenchyma and palisade cells. Furthermore, vascular bundles showed the presence of Al (Fig. 5), where the dye was more pronounced than in the rest of the leaf tissue. The main vein had an intense red colour in the parenchyma above the protoxylem, and similarly to the PCV staining, Al was found in the parenchyma cells of the xylem **[**see **Supporting Information]**.

## Discussion

### Phylogenetic implications

Based on our hydroponic experiments, *Symplocos* was found to accumulate Al when soluble Al was available to the plants. For only a small proportion of *Symplocos* species exact Al concentrations have been measured, and all show high levels of Al in their leaves (Table 1). The values for *S. chinensis* and *S. paniculata*, however, do not reflect natural conditions, and may therefore over- or underestimate the Al levels of plants in their natural environment. Nevertheless, the overall accumulation in *S. paniculata* was lower than in *S. chinensis*, which accumulated on average 8309 ± 282 mg kg^−^^1^ Al in its leaves after a 4-month hydroponic experiment with half the concentration used in our experiments (0.5 mM L^−1^ AlCl_3_) (Maejima *et al.*, 2014). *S. chinensis* is closely related to *S. paniculata* and both species have been suggested to be taxonomically merged to a single species, or at least form a separate phylogenetic clade (Wang *et al.*, 2004; Nooteboom, 2005).

**Table 1.** Mean foliar aluminium concentration of 13 *symplocos* species compiled from literature (see References) and the present study.

Species	Author	Al [mg·kg^−1^]	SD	References	Distribution
*S. ambangensis*	Noot.	16.719	1.12	Schmitt *et al*. 2016	SE-Asia
S. chinensis*	Jacq.	8.309	282	Maejima *et al*. 2014	Eastern Asia
*S. coreana*	Jacq.	14.766	n.a.	Watanabe et al., 2007	Eastern Asia
*S. crassipes*	Jacq.	33.883	n.a.	Metali, 2010	SE-Asia
*S. crataegoides*	Jacq.	6.56	n.a.	Chenery, 1948a, b	Central Asia
*S. lancifolia*	Jacq.	1.505	n.a.	Watanabe *et al.*, 2007	Eastern Asia
*S. microcalyx*	Jacq.	23.05	n.a.	Watanabe *et al.*, 2007	Eastern Asia
*S. myrtacea*	Jacq.	35.4	n.a.	Chenery, 1948a, b	Eastern Asia
*S.odoratissima*	Choisy ex Zoll.	23.383	9593	Schmitt *et al*., 2016	SE-Asia
*S. ophirensis*	C.B.Clarke	21.352	2802	Schmitt *et al*., 2016	SE-Asia
S. paniculata*	Miq.	4.125	3541	This study	Eastern Asia
*S. prunifolia*	Jacq.	1.85	n.a.	Watanabe *et al.*, 2007	Eastern Asia
*S. spicata*	Jacq.	72.3	n.a.	von Faber, 1925	Central Asia

Al concentration for *S. Chinensis* and *S. Paniculata* (asterisk) are based on hydroponic experiments and do not reflect natural conditions. Species distribution is based on the global biodiversity information facility (GBIF).

Given the basal phylogenetic position of *S. paniculata* within the genus (Wang *et al.*, 2004; Fritsch *et al.*, 2008) and based on earlier reports on the phylogeny of Al accumulation within angiosperms (Chenery, 1948b, 1949; Jansen *et al.*, 2002, 2003, 2004), this study suggests that Al accumulation characterizes all *Symplocos* species. Therefore, Al accumulation is not limited to tropical *Symplocos* species. In general, this trait is rare for temperate plants. An exception, however, is the Diapensiaceae family, which belongs to the same clade as Theaceae and Symplocaeae within the Ericales (Chenery, 1951; Jansen *et al.*, 2004).

### Al distribution within the plants

The variable Al concentrations in the tissues and organs most likely reflect the transport pathways of Al within the plant and show similar distribution patterns as naturally growing *Symplocos* trees in Indonesia (Schmitt *et al.*, 2016). Although the chemical form in which Al is taken up by the plant is unknown, the primary uptake mechanism appears to be transport via the transpiration stream, as the highest concentrations were observed in the leaves of the plants. The most commonly reported forms of Al complexes detected in the xylem sap of Al accumulators are Al citrate, malate, oxalate and fluorine, as these molecules are known to have a strong binding affinity to Al (Watanabe and Osaki, 2001; Flaten, 2002; Morita *et al.*, 2004; Satoh, 2006; Klug *et al.*, 2015). The root represents the only organ that is directly in contact with soluble Al, explaining the high concentrations within the root tissue. High concentrations of Al have also been found in the bark tissue of *S. paniculata*, indicating a possible participation of the phloem tissue in Al uptake. A similar interaction has been reported for *Camellia oleifera* (Zeng *et al.*, 2013) and is likely to occur as Al is known to have a high binding affinity to a variety of carbohydrates and ligands with carboxylate and phenolate functional groups (Miltner and Zech, 1998; Flaten, 2002). It is unclear whether the distribution of Al into the phloem follows a transport pathway similar to photosynthetic products in leaves, or is transported axially from the roots and then spreads radially from the stem xylem into the bark. Radial transport from the xylem into vessel associated parenchyma cells and subsequent transport to the bark tissue via the ray parenchyma is indicated by the staining methods applied and supports a potential interaction between xylem and phloem (Fig. 5). A positive correlation between Al and Ca was found for the seedlings, which was also reported for the tropical Al accumulators in Sulawesi (Schmitt *et al.*, 2016) and Sumatra (Masunaga *et al.*, 1998), suggesting a possible link of both metals in their transport pathway.

### Beneficial effect of Al

Whether or not Al has a beneficial effect on the growth of *S. paniculata* is complicated by the differences observed between seedlings and saplings. The seedlings did not seem to indicate a beneficial effect of Al on the growth of *S. paniculata*. None of the features measured (e.g. leaf area, leaf number, biomass, root–shoot ratio) showed a significant difference between the +Al and –Al seedlings. However, a tendency towards induced root growth could be found in the +Al seedlings, although no statistical significance was found for this trend.

The saplings, however, showed a pronounced difference between the –Al and +Al treatment. During the first two weeks in hydroponics, all saplings showed a stress reaction, indicated by leaf desiccation and partial leaf chlorosis at the leaf margins. Eventually all but one specimen of the –Al treatment died, whereas the +Al treatment saplings recovered, flushed new leaves and produced new side branches. Furthermore, the +Al saplings significantly increased their root biomass, which was indicated by an increase in the root–shoot ratio. A similar increase in fine roots and increased biomass due to Al application was reported by Watanabe *et al.* (2005a) in saplings of *Melastoma malabathricum*. Fung *et al.* (2008) also reported a beneficial, stimulatory effect on the roots and an enhanced nutrient uptake in *Camellia sinensis* seedlings.

The difference in growth and plant mortality between the seedlings and saplings remain unclear. One explanation could be the origin of the seeds and the saplings, which were taken from different populations. The genetic variability could affect the growth of the plants and their susceptibility when transferred from a soil substrate to hydroponic condition. The potential impact of genetic variability on plants growing in hydroponics has been reported for *Sorghum bicolor* and *Fagopyrum esculentum* (Jordan *et al.*, 1979; Klug *et al.*, 2015), but may not explain the subsequent death of the –Al plants. Furthermore, earlier growth conditions between our seedlings and saplings differed, as the saplings were grown in a greenhouse, experiencing mild winter temperatures in contrast to the seedlings, which were germinated at ambient winter temperatures. The harsh winter conditions for the seedlings might have been beneficial for the hydroponics experiment, while the saplings obtained from a nursery were probably more sensitive to environmental changes. It is also possible that developmental differences in nutrient requirements contributed to the differences in our hydroponic experiment between seedlings and saplings.

Before we conducted our experiments, *S. paniculata* plants were grown in peat soil with a relatively low pH (ca. 5.5). Despite the fact that the species investigated is a temperate shrub, the natural distribution of *Symplocos* is in tropical to subtropical environments, mostly reflecting biomes with a low soil pH. Therefore, soil acidity below 5.5 cannot be regarded as a potential harm for the *S. paniculata* plants in our experimental setup, because the plants are naturally adapted to a low soil pH status (Brunner and Sperisen, 2013). Furthermore, a low soil pH is needed to ensure the availability of Al^3+ ^to the plants.

The only difference between the +Al and –Al treatments was the added 1 mM AlCl_3_. Thus, a possible explanation for the death of the –Al saplings could be the stress reaction induced by the hydroponic condition together with an impaired nutrient uptake. The limited availability of phosphorous (P) in the nutrient solution could also be a potential explanation for induced root growth in the +Al treatment, which is known to occur if plants experience a P-deficient substrate (Kochian *et al.*, 2004; Kochian, 2012). Watanabe and Osaki (2001) reported a link between Al application and P nutrition in *M. malabathricum*, which did not indicate P as primary reason for growth enhancement. Konishi *et al.* (1985) observed a stimulatory effect of Al on the P-uptake in tea plants, which could also confirm our findings for the +Al treatment. A potential precipitation of Al with P, which reduces the P-availability, could be suggested (Watanabe *et al.*, 2005a), and may account for the increased root growth observed. This scenario would explain the growth of the +Al treatment sapling as an indirect effect because of the added Al, which would subsequently precipitate the P and reduce its availability. However, the –Al treatment had the same nutrient solution with the same P concentration. Thus, the enhanced root growth due to P deficiency would also have been observed in the –Al treatment, which means that P availability cannot be regarded as the primary and only reason for the high mortality of the –Al saplings.

## Conclusions

Our hydroponic experiments indicate that *S. paniculata* has the capacity to accumulate Al in its aboveground tissues. Al accumulation in this species appears to be facultative and the beneficial effect of Al on its growth is complicated by the different response to Al between seedlings and saplings. Variation in Al levels across different organs and tissues in *S. paniculata* is similar to tropical species within this genus, with the highest Al concentrations occurring in leaves and bark tissue.

## Sources of Funding

This work was funded by the Juniorprofessuren-Programm of the Ministry for Science, Research and the Arts of Baden-Württemberg, Germany.

## Contributions by the Authors

M.S. and S.J. planned the hydroponic experiments. M.S. conducted the hydroponic experiments, the elemental analysis and the anatomical observations. T.W. gave critical input to the hydroponic experiments and the setup. All authors contributed substantially to the writing of the manuscript.

## Conflict of Interest Statement

None declared.

## Supplementary Material

Supplementary Data

## Supporting Information

The following additional information is available in the online version of this article —

**Figure S1**. Hydroponic setup for the growth experiment with 16 specimens of *Symplocos paniculata* saplings in the greenhouses of the Botanical Garden at Ulm University. (A) Setup at the beginning of the experiment. (B) Setup at the end of the experimental phase. Except for the tallest plant (arrow), the –Al plants on the left side were all dead, whereas the +Al plants were resprouting and healthy. The grey cover plates prevented the growth of algae.

**Figure S2**. Magnified cross sections of the central leaf vein of *Symplocos paniculata* stained with two different techniques to visualize Al in the tissue in plants that were growing in a nutrient solution containing 1 mM AlCl_3_ for 4 months. Black arrows show the presence of Al in the ray cells of the xylem tissue of the central vein. (A) Aluminon. (B) Pyrocatechol-violet. (C) Staining with aluminon in a plant growing without Al. Scale bar  = 100 µm

**Table S1**. Elemental concentrations of various tissues of *Symplocos paniculata* growing in hydroponic solutions for 4 months (*n*  = 8 saplings per treatment) and 2 months (*n*  = 10 seedlings per treatment). Plants in the +Al-treatment were given 1 mM AlCl_3_. All values are given in mean mg kg^−^^1^ dry mass (± SD). New roots were only formed in the +Al seedling plants and not in the seedlings.
