# Supplementary material for: The effects of aluminium on plant growth in a temperate and deciduous aluminium accumulating species
Source: AoB Plants. 2016 Oct 26;8:plw065. doi: 10.1093/aobpla/plw065 (PMC5091896; doi:10.1093/aobpla/plw065)
Supplement: Supplementary Data [file supp_8_plw065_index.html]

The effects of aluminium on plant growth in a temperate and deciduous aluminium accumulating species — Supplementary Data 

# The effects of aluminium on plant growth in a temperate and deciduous aluminium accumulating species

## Supplementary Data

files

- Supplementary Data - docx file
