# Supplementary material for: The effects of aluminium on plant growth in a temperate and deciduous aluminium accumulating species
Source: AoB Plants. 2016 Oct 26;8:plw065. doi: 10.1093/aobpla/plw065 (PMC5091896; doi:10.1093/aobpla/plw065)
Supplement: Supplementary Data [file supp_plw065_aobplants-16072-s01.docx]

**SUPPORTING INFORMATION**

**Supplementary Figure 1.** Hydroponic setup for the growth experiment with 16 specimens of *Symplocos paniculata* saplings in the greenhouses of the Botanical Garden at Ulm University. **A**: Setup at the beginning of the experiment; **B**: Setup at the end of the experimental phase. Except for the tallest plant (arrow), the –Al plants on the left side were all dead, whereas the +Al plants were resprouting and healthy. The grey cover plates prevented the growth of algae.


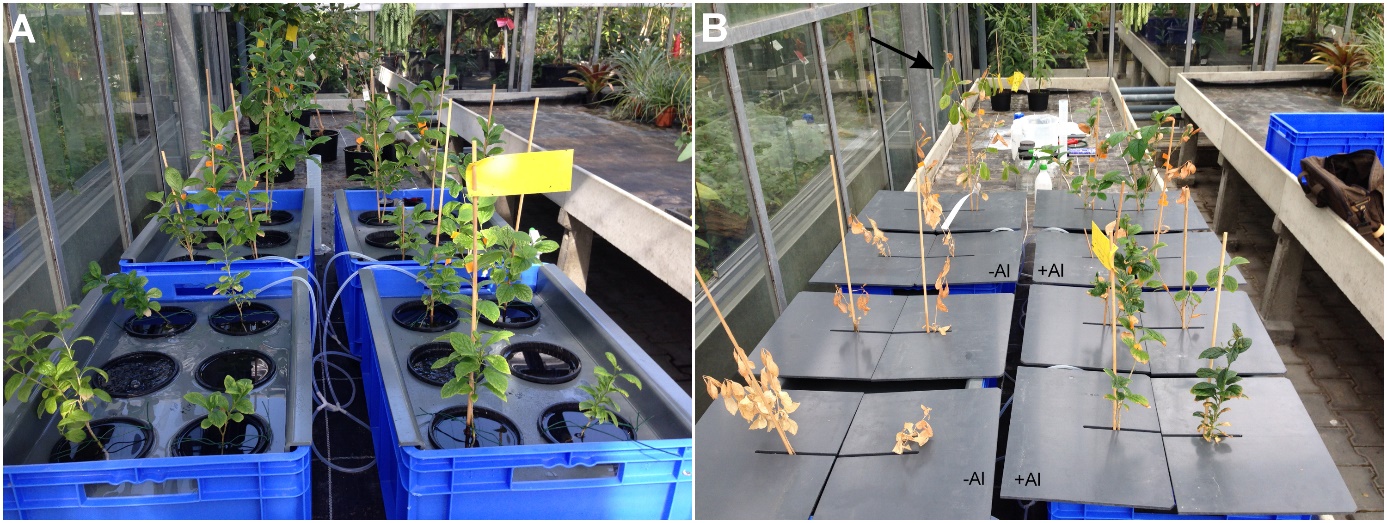


**Supplementary Figure 2.** Magnified cross sections of the central leaf vein of *Symplocos paniculata* stained with 2 different techniques to visualize Al in the tissue in plants that were growing in a nutrient solution containing 1 mM AlCl_3_ for 4 months. Black arrows show the presence of Al in the ray cells of the xylem tissue of the central vein. **A**: Aluminon. **B**: Pyrocatechol-violet. **C**: Staining with aluminon in a plant growing without Al. Scale bar = 100 µm


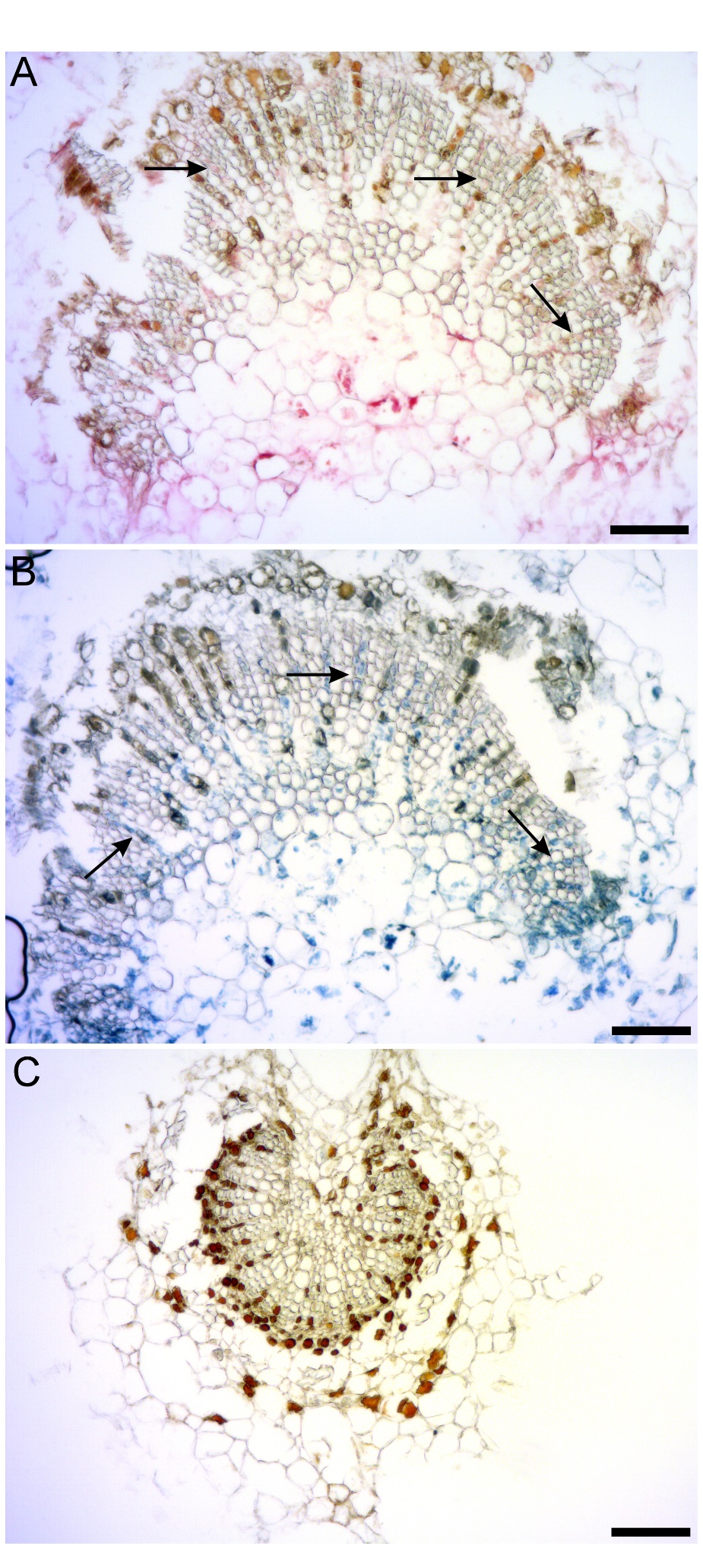


**Supplementary Table 1.** Elemental concentrations of various tissues of *Symplocos paniculata* growing in hydroponic solutions for 4 months (n = 8 saplings per treatment) and 2 months (n = 10 seedlings per treatment). Plants in the +Al-treatment were given 1 mM AlCl_3_. All values are given in mean mg·kg^-1^ dry mass (± SD). New roots were only formed in the +Al seedling plants and not in the seedlings.

| **Saplings** |  |  |  |  |  |  |
| --- | --- | --- | --- | --- | --- | --- |
| **Treatment** | **Tissue** | **Al [mg·kg^-1^]** | **Ca [mg·kg^-1^]** | **Fe [mg·kg^-1^]** | **K [mg·kg^-1^]** | **Mg [mg·kg^-1^]** |
| +Al | wood | 1,618 ± 842 | 1,011 ± 194 | 120 ± 58 | 5,365 ± 1,393 | 818 ± 121 |
| +Al | bark | 3,670 ± 1.844 | 7,999 ± 1,627 | 192 ± 32 | 15,402 ± 3,461 | 2,977 ± 392 |
| +Al | leaf | 4,290 ± 4,025 | 9,216 ± 2,092 | 185 ± 75 | 21,971 ± 2,338 | 4,315 ± 713 |
| +Al | root (old) | 6,387 ± 1,894 | 981 ± 237 | 17,193 ± 7,803 | 5,993 ± 3,349 | 868 ± 394 |
| +Al | root (new) | 12,936 ± 5,294 | 1,220 ± 339 | 26,875 ± 33,762 | 15,975 ± 7,279 | 2,017 ± 964 |
|  |  |  |  |  |  |  |
| -Al | wood | 46 ± 21 | 3,238 ± 927 | 135 ± 80 | 9,157 ± 3,046 | 1,926 ± 767 |
| -Al | bark | 155 ± 30 | 9,820 ± 3,227 | 413 ± 298 | 12,363 ± 3,539 | 3,756 ± 441 |
| -Al | leaf | 163 ± 82 | 8,285 ± 1,184 | 253 ± 136 | 26,161 ± 2,971 | 4,483 ± 438 |
| -Al | root | 196 ± 168 | 1,445 ± 280 | 31,698 ± 13,774 | 2,798 ± 1,191 | 578 ± 292 |
|  |  |  |  |  |  |  |
| **Seedlings** |  |  |  |  |  |  |
| **Treatment** | **Tissue** | **Al [mg·kg^-1^]** | **Ca [mg·kg^-1^]** | **Fe [mg·kg^-1^]** | **K [mg·kg^-1^]** | **Mg [mg·kg^-1^]** |
| +Al | wood | 1,038 ± 357 | 700 ± 147 | 67 ± 17 | 3,206 ± 699 | 661 ± 119 |
| +Al | bark | 3,054 ± 419 | 8,753 ± 2,227 | 454 ± 274 | 10,729 ± 982 | 2,302 ± 271 |
| +Al | leaf | 4,107 ± 1,474 | 8,996 ± 2,347 | 179 ± 41 | 26,417 ± 4,122 | 3,894 ± 739 |
| +Al | root | 3,749 ± 1,204 | 1,357 ± 293 | 2,644 ± 1,437 | 5,764 ± 1,099 | 1,589 ± 546 |
|  |  |  |  |  |  |  |
| -Al | wood | 136 ± 32 | 620 ± 92 | 156 ± 37 | 2,673 ± 642 | 612 ± 148 |
| -Al | bark | 481 ± 197 | 8,006 ± 1,997 | 609 ± 185 | 9,893 ± 1,303 | 2,394 ± 445 |
| -Al | leaf | 456 ± 243 | 9,738 ± 2,360 | 336 ± 297 | 24,592 ± 3,746 | 4,498 ± 733 |
| -Al | root | 364 ± 153 | 1,471 ± 328 | 6,668 ± 4,242 | 4,771 ± 831 | 2,394 ± 445 |
